# Novel Experimental Dietary Supplementations to Combat Metabolic Alterations in Diabetes

**DOI:** 10.3390/nu17111816

**Published:** 2025-05-27

**Authors:** Joana Rossell, Josep Julve

**Affiliations:** 1 Center for Biomedical Research on Diabetes and Associated Metabolic Diseases (CIBERDEM), Instituto de Salud Carlos III, 28028 Madrid, Spain; joanarossell@gmail.com; 2 Institut de Recerca Sant Pau (IR SANT PAU), Sant Quintí 77-79, 08041 Barcelona, Spain

Metabolic diseases such as diabetes mellitus (DM), metabolically associated liver disease, obesity, and atherosclerosis are becoming highly prevalent worldwide, contributing to an increased mortality. This increase is mainly attributed to their association with other diseases, particularly cardiovascular diseases (CVDs), a leading cause of death, according to the World Health Organization. Dietary manipulations—alone or combined with pharmacological approaches—should be one of the main strategies for addressing metabolic diseases, especially at the earlier stages. Accumulating evidence suggests that adherence to healthy dietary patterns is crucial for managing cardiometabolic diseases. The Mediterranean diet has long been known to protect against metabolic stress and reduce the risk of related diseases. This diet is mainly characterized by a high consumption of edible plant components, including virgin olive oil, fruits, vegetables, legumes, nuts, and unrefined grains. Herein, on behalf of the Special Issue, “Dietary Manipulations: Advances in Metabolism Diseases”, we are pleased to present several novel studies exploring the metabolic benefits of consuming nuts, one of the main edible plant food components of the Mediterranean diet, in both clinical and experimental settings. The metabolic benefits of other experimental dietary supplementations on DM in experimental settings are also addressed.

## 1. Nuts: Metabolic Effects of One of the Main Plant Components of the Mediterranean Diet

The consumption of nuts, a main component of the Mediterranean diet [1], has been shown to improve various aspects of human health related to metabolic diseases. In fact, it has been associated with a reduced risk of CVD [2,3], enhanced management of DM and obesity [4], improvements in blood pressure, lipid profile, and inflammatory markers, and a reduction in atherogenic cardiovascular disease [5].

Regarding lipid metabolism, nuts—particularly walnuts—have been linked to a reduction in both low-density lipoproteins (LDLs) and total cholesterol [6], which contribute to a reduced risk for CVD. Notably, the recent advent of advanced nuclear magnetic resonance-based approaches has revealed hidden changes in remnant proatherogenic lipoproteins, primarily derived from very-low-density lipoproteins (VLDLs) and intermediate-density lipoproteins (IDLs), thereby overcoming the technical limitations of traditional lipid measurements and providing additional insights into CVD risk [7]. In this context, a study by Nijssen K. M. R. et al. [8] investigated the influence of long-term (16 weeks) consumption of mixed nuts (walnuts, hazelnuts, pistachios, and cashews) by a small cohort of healthy adult subjects (aged 60–70 years) who were overweight/obese (25–35 kg/m^2^) on the advanced characteristics of different circulating lipoprotein subclasses. Specifically, mixed nut consumption significantly reduced the proatherogenic profile of the circulating concentrations across a broad range of apolipoprotein (apo)B-containing lipoproteins, as indicated by concomitant reductions in plasma VLDL, IDL, and large LDL concentrations. Interestingly, the triglyceride content of all the lipoprotein subclasses was also favorably reduced in the subjects who consumed nuts. Altogether, the observed improvements in the lipoprotein profile of treated subjects suggest that long-term consumption of mixed nuts could attenuate CVD risk. However, further research may be required to directly assess the potential contribution, if any, of the improved lipoprotein profile relative to other traditional lipid variables, as clinical CVD outcomes were not assessed in this interventional cohort.

Beyond the well-known beneficial effects of nuts, nut by-products, such as peanut shells, which are rich in antioxidants like flavonoids, phenolic compounds, and lignans, may also be of interest as a supplement for targeting diseases like DM. DM is defined as a chronic metabolic disorder that is often linked to disturbances in insulin signaling, as well as carbohydrate and lipid metabolism [9]. Deshmukh H. et al. [10] evaluated the potential positive impact of dietary peanut shell extract supplementation on mitochondrial dysfunction in different target tissues—brain, liver, and white adipose tissue—of a diabetic mouse model (db/db mice). The treated mice exhibited an ameliorated mitochondrial function, as shown by the improved gene expression/protein abundance profile of key molecular targets involved in mitochondrial biogenesis and dynamics in the above-mentioned target tissues. Specifically, dietary supplementation with peanut shell extract attenuated the increased tissue expression observed in untreated db/db mice of both DRP1, a molecular marker involved in mitochondrial fission, and PINK1, a marker for mitophagy. These changes were also accompanied by concomitant elevations in the gene and protein abundance of NRF2, as well as reductions in the abundance of TNFα in the same target tissues of treated mice. However, several parameters, such as mitochondrial functionality, tissue antioxidant enzymes, e.g., hemoxygenase-1 and superoxide dismutase-2, whose expression is modulated by NRF2, and reactive oxygen species production, were not assessed in this study. Therefore, further analysis may be necessary to confirm the enhanced mitochondrial function and oxidative stress.

## 2. Further Metabolic Effects of Experimental Dietary Supplementations in Diabetes

In the study by Huang C.-H. et al. [11], the authors investigated the impact of alkaline reduced water (ARW) supplementation on glucose and lipid metabolism in both non-diabetic and diabetic rats fed a high-fat diet. ARW, also known as alkaline ionized water or electrolyzed reduced water, is produced through the electrolysis of water. The pH range of the ARW used in this study was approximately 10.0–10.5, which is higher than that of regular drinking water (with pH values not exceeding 8.5, according to World Health Organization and Environmental Protection Agency recommendations). While some studies have suggested potential health benefits, including the ability to reduce oxidative stress and promising use as an anti-diabetic treatment [12], other research points to possible harmful effects of ARW, especially at a pH of 10 or more, advising individuals with poor kidney function to refrain from consuming ARW [13]. In this study, diabetic rats drinking ARW demonstrated significantly reduced fasting glucose concentrations. Under non-diabetic conditions, drinking ARW also reduced the hyperinsulinemia observed in the regular water group. Remarkably, ARW administration led to significant reductions in circulating total cholesterol concentrations, mainly due to concomitant decreases in the concentrations of VLDL and LDL cholesterol in the non-diabetic rats. Consistently, circulating triglyceride concentrations across all lipoprotein classes, including high-density lipoproteins (HDLs), were also reduced in ARW-treated, non-diabetic rats compared with non-treated, non-diabetic rats. Intriguingly, ARW failed to induce the same changes in the lipid profile of the diabetic rats, which showed no differences compared to the profile of the non-diabetic rats. Instead, ARW lowered the plasma HDL cholesterol levels in the diabetic rats, in contrast to the non-treated, diabetic rats. The hepatic effects of ARW were also investigated. The non-diabetic rats drinking ARW accumulated significantly more cholesterol in the liver than the non-diabetic rats receiving regular water, suggesting hepatic retention. Consistently, the fecal excretion of cholesterol was reduced in this group. Conversely, ARW increased the levels of fecal triglyceride content in diabetic rats, which showed no differences in either hepatic or fecal cholesterol content. Moreover, the antioxidative potential of ARW supplementation was only demonstrated in the treated, non-diabetic rats. Although the reason for the disparity in lipid effects induced by ARW between the diabetic and non-diabetic rats remains unclear, it may be attributed to metabolic differences between the two groups.

Purine and pyrimidine nucleotides are fundamental components of nucleic acids; however, they also play pivotal roles in modulating different metabolic processes, acting as chemical energy carriers, signaling molecules, or constituents of coenzymes [14]. In this regard, recent research has suggested the potential therapeutic use of preformed purines and pyrimidines to favorably influence metabolic disturbances in target tissues. In a study by Song L. et al. [15], the impact of nucleotide administration on hepatic insulin signaling, which was distorted in in vitro conditions mimicking insulin resistance induced by palmitic acid, was investigated. Supplementation with exogenous nucleotides in palmitic acid-treated HepG2 cells increased glucose consumption and positively influenced intracellular glycogen content. Furthermore, these findings were accompanied by a favorable rewire of insulin sensitivity, as well as reduced oxidative stress and inflammation in the treated cells. Future research may be needed to elucidate the underlying mechanisms and further explore the favorable effects of nucleotide intervention in vivo, using an experimental model of liver disease.

## 3. Dietary Counselling: A Key Intervention for Preventing Diabetic Retinopathy

A comprehensive review by Yang C. et al. [16] examined the negative influence of persistent hyperglycemia on the development of diabetic retinopathy (DR). In DM, chronic elevations of blood glucose may cause tissue damage by enhancing oxidative stress, inflammation, and the production of advanced glycation end products. In the case of the retina, tissue remodeling is further exacerbated by the increased production of vascular endothelial growth factor, which may eventually lead to irreversible visual impairment. There is no specific therapy to prevent the development of DR, with current therapies primarily targeting the terminal stages of this condition. Thus, early prevention of DR, which can achieved through glycemic optimization and dietary counseling, is particularly critical. This review primarily describes the main risk factors of DR and delves into the pathophysiological and molecular mechanisms involved in its development, which is typically caused by diets rich in sucrose. A diet rich in carbohydrates and simple sugars produces constant glucose elevations and, concomitantly, an increase in the rest of factors contributing to DR. Prolonged exposure to these factors is one of the main risk factors for developing DR. Importantly, this review underscores the crucial role of dietary modifications and how optimal blood glucose control prevents the progress of DR, ultimately avoiding permanent vision loss.

## 4. Conclusions

We believe that the studies featured in this Special Issue will serve as a valuable resource for researchers focused on the evaluation of nutritional interventions that positively influence the altered metabolic features of T2D, potentially paving the way for the development of novel therapeutic strategies. 

## Abbreviations

The following abbreviations are used in this manuscript:

ARW	Alkaline Reduced Water
CVD	Cardiovascular Disease
DM	Diabetes Mellitus
DR	Diabetic Retinopathy
HDL	High-Density Lipoproteins
IDL	Intermediate-Density Lipoproteins
LDL	Low-Density Lipoproteins
VLDL	Very-Low-Density Lipoproteins
